# Rapid detection of *Acinetobacter baumannii* and molecular epidemiology of carbapenem-resistant *A. baumannii* in two comprehensive hospitals of Beijing, China

**DOI:** 10.3389/fmicb.2015.00997

**Published:** 2015-09-23

**Authors:** Puyuan Li, Wenkai Niu, Huan Li, Hong Lei, Wei Liu, Xiangna Zhao, Leijing Guo, Dayang Zou, Xin Yuan, Huiying Liu, Jing Yuan, Changqing Bai

**Affiliations:** ^1^Department of Respiratory and Critical Care Diseases, 307th Hospital of Chinese People’s Liberation ArmyBeijing, China; ^2^Institute of Disease Control and Prevention, Academy of Military Medical SciencesBeijing, China; ^3^Department of Clinical Laboratory, 309th Hospital of Chinese People’s Liberation ArmyBeijing, China

**Keywords:** *Acinetobacter baumannii*, LAMP, rapid diagnosis, carbapenem resistance, molecular epidemiology

## Abstract

*Acinetobacter baumannii* is an important opportunistic pathogen associated with a variety of nosocomial infections. A rapid and sensitive molecular detection in clinical isolates is quite needed for the appropriate therapy and outbreak control of *A. baumannii*. Group 2 carbapenems have been considered the agents of choice for the treatment of multiple drug-resistant *A. baumannii*. But the prevalence of carbapenem-resistant *A. baumannii* (CRAB) has been steadily increasing in recent years. Here, we developed a loop-mediated isothermal amplification (LAMP) assay for the rapid detection of *A. baumannii* in clinical samples by using high-specificity primers of the *bla*_OXA-51_ gene. Then we investigated the OXA-carbapenemases molecular epidemiology of *A. baumannii* isolates in two comprehensive hospitals in Beijing. The results showed that the LAMP assay could detect target DNA within 60 min at 65°C. The detection limit was 50 pg/μl, which was about 10-fold greater than that of PCR. Furthermore, this method could distinguish *A. baumannii* from the homologous *A. nosocomialis* and *A. pittii*. A total of 228 positive isolates were identified by this LAMP-based method for *A. baumannii* from 335 intensive care unit patients with clinically suspected multi-resistant infections in two hospitals in Beijing. The rates of CRAB are on the rise and are slowly becoming a routine phenotype for *A. baumannii.* Among the CRABs, 92.3% harbored both the *bla*_OXA-23_ and *bla*_OXA-51_ genes. Thirty-three pulsotypes were identified by pulsed-field gel electrophoresis, and the majority belonged to clone C. In conclusion, the LAMP method developed for detecting *A. baumannii* was faster and simpler than conventional PCR and has great potential for both point-of-care testing and basic research. We further demonstrated a high distribution of class D carbapenemase-encoding genes, mainly OXA-23, which presents an emerging threat in hospitals in China.

## Introduction

*Acinetobacter baumannii* is an important opportunistic pathogen associated with a variety of nosocomial infections, such as ventilator-associated pneumonia, central line-associated bloodstream infections, urinary tract infections, surgical-site infections, and other types of wound infections, especially in intensive care units (ICUs; Peleg et al., 2008). This organism is well adapted to hospital environments, being capable of continually spreading to new patients and making itself a nosocomial pathogen of particular clinical concern and a public health threat (Dijkshoorn et al., 2007).

In recent years, multidrug-resistant *A. baumannii* (MDR-AB) isolates have been increasingly reported worldwide. MDR-AB strains are associated with an enhanced risk of mortality and prolonged durations of hospitalization (Kempf and Rolain, 2012; Antunes et al., 2014; Lemos et al., 2014). Carbapenems, mainly imipenem (IPM) and meropenem (MPM), have been used to treat MDR-AB infections (Perez et al., 2007). However, the incidence of carbapenem resistance in *A. baumannii* is growing steadily in China and many other countries (Poirel and Nordmann, 2006; Tiwari et al., 2012a,b). According to the CHINET 2010 year reports, the resistant to IPM and MPM in *A. baumannii* isolates was 62.3% and 63.8%, respectively (Xi et al., 2012). However, few data are available regarding the molecular epidemiology and antibiotic resistance of *A. baumannii* infections in hospitals in Beijing, China.

The accurate and rapid identification of *A. baumannii* is critical for appropriate infection control in hospital settings. To date, the most common and widespread detection methods include characterization via a phenotypic system and commercial phenotypic methods (e.g., the VITEK 2 system [Biomerieux] and the API 20 NE system) or DNA-based testing such as PCR (e.g., 16S rRNA gene amplification), which have been used to successfully identify most *Acinetobacter* species. However, there are some limitations in these methods (Janda and Abbott, 2007; Karah et al., 2011; Álvarez-Buylla et al., 2012). For example, several days are needed for incubation, and the laboratory diagnosis of *A. baumannii* is actually for that of the *A. calcoaceticus*–*A. baumannii* complex (ABC), which includes *A. calcoaceticus*, *A. baumannii*, *A. pittii* (three species), and *A. nosocomialis* (species 13TU; Peleg et al., 2008). These bacterial species are different in terms of symptomatology, dissemination patterns, mechanisms of antibiotic resistance, and epidemiology (Golanbar et al., 2011; Chiang et al., 2012).

The loop-mediated isothermal amplification (LAMP) method, which is based on autocycling strand displacement DNA synthesis in the presence of Bst DNA polymerase, can be used to amplify target DNA with high specificity (as four or six specific primers that recognize six or eight different sequences on the DNA target) under isothermal conditions in less than 60 min (Notomi et al., 2000). LAMP is highly sensitive and able to detect DNA at as few as six copies in the reaction mixture, and less prone to the presence of irrelevant DNA than PCR (Notomi et al., 2000). This novel method has been developed widely for the detection of numerous pathogens, including influenza A subtypes H1N1 (Nakauchi et al., 2011), H5N1 (Dinh et al., 2011), H7N9 (Nakauchi et al., 2014), as well as *Mycoplasma pneumoniae* (Gotoh et al., 2013), *Mycobacterium tuberculosis* (Kumar et al., 2014), severe acute respiratory syndrome virus coronavirus (Poon et al., 2004), and human immunodeficiency virus (Zeng et al., 2014).

In present study, we describe a LAMP method for the rapid detection of *A. baumannii* in clinical samples targeting the *bla*_OXA-51_ gene, which was based on visual testing. We also investigated molecular-epidemiology and antibiotic-resistance profiles, and detected several common oxacillinase genes from *A. baumannii* isolates obtained at two comprehensive hospitals in Beijing, China.

## Materials and Methods

### Bacterial Strains, Antimicrobial Susceptibility Testing, and Pulsed-Field Gel Electrophoresis (PFGE)

A total of 34 strains representing 9 *Acinetobacter* species (2 *A. baumannii* strains and 10 other *Acinetobacter* strains) and 22 non-*Acinetobacter* species used in this study to develop the LAMP assays. Three hundred and fifty-five clinical sputum samples and nasopharyngeal swabs were obtained from the ICU hospitalized patients with clinically suspected multi-resistant infections in 307th (Affiliated Hospital of Academy of Military Medical Sciences), and the 309th Hospital of PLA in China. Pertinent information and the source of all strains are listed in **Table 1**. The species identification were identified by the Vitek 2 system (Biomerieux Vitek, Inc., Hazelwood, MO, USA). *A. baumannii* bacteria were grown overnight at 37°C in Luria-Bertani (LB) broth, while non-*Acinetobacter* species were cultured at 37°C in brain heart infusion (BHI) broth overnight.

**Table 1:** Bacteria strains used in this study.

Bacteria strains	Source	Strain type
***Acinetobacter* species**		
*A. baumannii*	CGMCC	Reference strain
*A. baumannii* ATCC 22933	CICC	Reference strain
*A. calcoaceticus*	CGMCC	Reference strain
*A. nosocomialis*	CGMCC	Reference strain
*A. haemolyticus*	CGMCC	Reference strain
*A. Pittii*	CICC	Reference strain
*A. johnsonii*	CGMCC	Reference strain
*A. junii*	CGMCC	Reference strain
*A. calcoaceticus*	The Microorganism Center^a^	Reference strain
*A. lwoffii*	The Microorganism Center (three strains)	Reference strain
**Non-*Acinetobacter* species**		
*Bacillus megatherium* 4623	The Microorganism Center	Reference strain
*Beta-haemolytic streptococcus* group A CMCC32213	The Microorganism Center	Reference strain
*Bordetella pertussis* ATCC 18530	The Microorganism Center	Reference strain
*Brucella suis* 3572	The Microorganism Center	Reference strain
*Corynebacterium diphtheria* CMCC38001	The Microorganism Center	Reference strain
*Enteropathogenic Escherichia coli* 2348	The Microorganism Center	Reference strain
*Enterotoxigenic E. coli* 44824	The Microorganism Center	Reference strain
*E. coli* ATCC25922	The Microorganism Center	Reference strain
*Mycobacterium tuberculosis* 8362	The Microorganism Center	Reference strain
*Neisseria meningitides* group B CMCC29022	The Microorganism Center	Reference strain
*Pseudomonas aeruginosa* ATCC27853	The Microorganism Center	Reference strain
*Salmonella aberdeen* 9264	The Microorganism Center	Reference strain
*Salmonella enteritidis* 50326-1	The Microorganism Center	Reference strain
*Stenotrophomonas maltophilia* 3859	The Microorganism Center	Reference strain
*Shigella flexneri* 4536	The Microorganism Center	Reference strain
*Shigella sonnei* 2531	The Microorganism Center	Reference strain
*Staphylococcus aureus* 2740	The Microorganism Center	Reference strain
*Vibrio carchariae* 5732	The Microorganism Center	Reference strain
*Vibrio cholera* 3802	The Microorganism Center	Reference strain
*Vibrio parahaemolyticus* 5474	The Microorganism Center	Reference strain
*Yersinia enterocxolitica* 1836	The Microorganism Center	Reference strain
*Yersinia pestis* 2638	The Microorganism Center	Reference strain

*CGMCC, China General Microbiological Culture Collection Center; CICC, China Center of Industrial Culture Collection. ^a^The Microorganism Center of the Institute of Disease Control and Prevention of the Academy of Military Medical Sciences*.

Antibiotic susceptibility testing was conducted by disk diffusion in accordance with the guidelines of the Clinical and Laboratory Standards Institute and was confirmed by the Vitek 2 System with the following antibiotics: ampicillin, ampicillin/sulbactam, aztreonam, ciprofloxacin, ceftazidime, ceftriaxone, cefepime, gentamycin, levofloxacin, IPM, MPM, piperacillin/tazobactam, piperacillin, tobramycin, and sulfamethoxazole. *Pseudomonas aeruginosa* ATCC 27853 was used as a control.

Genomic DNA was prepared in agarose plugs, and digested with the restriction enzyme *Apa*I (New England BioLabs, Beverly, MA, USA). The DNA restriction fragments were separated using a CHEF-DRIII apparatus (Bio-Rad Laboratories, Richmond, CA, USA). Gel images were analyzed using BioNumerics software, version 6.01 (Applied-Maths, Belgium). The interpretation of gels was performed by visual inspection using the criteria of Tenover et al. (1995).

### Preparation of Isolated DNA

Genomic DNA from *Acinetobacter* species was prepared using the boiling method described by Olive and Bean (1999), with some modifications. Briefly, colonies of each isolate were picked from LB plates and suspended in 100 μl of ddH_2_O. The bacterial suspensions were boiled at 95°C for 15 min, centrifuged at 12,000 rpm for 5 min, and the supernatants were collected and transferred to fresh Eppendorf tubes as templates for use in LAMP and PCR assays. To determine the sensitivity and specificity of the LAMP assays, bacterial genomic DNA was extracted from *A. baumannii* using the Wizard^®^ Genomic DNA Purification Kit A1125 (Promega, Madison, WI, USA). The DNA concentration was measured by using OD260 measurements (ND-1000 spectrophotometer, NanoDrop Technologies, Inc, Wilmington, DE, USA) and prepared by serial 10-fold dilutions to yield concentrations ranging from 500 ng/μl to 0.05 pg/μl.

### Primer Design for LAMP Assay

The sequence of *bla*_OXA-51_ (GenBank No. DQ385606.1) was downloaded from the NCBI GenBank database and used to design *bla*_OXA-51_-specific LAMP primers. The sequence was further analyzed by Primer Explorer software (version 4; http://www.primerexplorer.jp/lamp/), and five sets of primers were designed, including the outer forward primer (F3), the outer backward primer (B3), the forward inner primer (FIP), the backward inner primer (BIP), and additional loop primers (loops F and B), as shown in **Table 2**.

**Table 2:** Sequence of primers used for specific amplification of *bla*_OXA-51-like_ and the OXA carbapenemases PCR detection.

Primer	Primer type	Sequence (5′–3′)
OXA-4F3	Forward outer	CTTATATAGTGACTGCTAATCCAA
OXA-4B3	Backward outer	ATTAAGCATTTTGAAGGTCGA
OXA-4FIP	Forward inner	ACCCGTAGTGTGTACTTCGTTAAATTTTTACAGCGCTTCAAAATCTGA
OXA-4BIP	Backward inner	TTAGTTATCCAACAAGGCCAAACTTTTTAGCAGGTACATACTCGGTC
OXA-4LB	Loop forward	AAAGCTATGGTAATGATCTTGCTCG
*bla*_OXA-23-F_	Forward	ATGAATAAATATTTTACTTG
*bla*_OXA-23-R_	Reverse	TTAAATAATATTCAGCTGTT
*bla*_OXA-24-F_	Forward	ATGAAAAAATTTATACTTCCTATATTCAGC
*bla*_OXA-24-R_	Reverse	TTAAATGATTCCAAGATTTTCTAGC
*bla*_OXA-51-like-F_	Forward	TAATGCTTTGATCGGCCTTG
*bla*_OXA-51-like-R_	Reverse	CTATAAAATACCTAATTKTTCTAA
*bla*_OXA-58-F_	Forward	ATGAAATTATTAAAAATATTGAGT
*bla*_OXA-58-R_	Reverse	ATAAATAATGAAAAACACCCAA

### LAMP Reaction and Product Detection

LAMP reactions were performed in a total volume of 25 μl and contained 12.5 μl reaction mixtures (DNA Amplification Kit; Eiken Chemical Co., Ltd., Tochigi, Japan), 2.6 μl primer mixture (40 pmol for FIP and BIP, 20 pmol for LF and LB, and 5 pmol for F3 and B3), and 1 μl of *Bst* polymerase (eight units, New England BioLabs, Ipswich, MA, USA). Finally, 1-μl DNA of genomic template DNA (the concentration is above 50 ng/μl) was added to the reaction tubes. Reaction was performed in a LA-320CE instrument (Eiken Chemical Co., Ltd., Tochigi, Japan) at 65°C for 60 min and stopped by heating to 80°C for 5 min, according to the manufacturer’s instructions. The LA-320CE instrument can monitor the turbidity of real-time LAMP products through spectrophotometric analysis by recording the optical density at 650 nm every 6 s with the help of a Loopamp real-time turbidimeter. In addition, a visual color-change detection method was employed. Briefly, 1 μl of fluorescence-detection reagent (Eiken Chemical Co., Ltd., Tochigi, Japan) was added to each 25 μl LAMP-reaction mixture prior to initiating the reactions. Positive reactions were identified by a green color change, while negative reactions remained orange in color. The color change could be observed by the naked eye under natural light, or with the aid of ultraviolet light excitation at 365 nm.

To compare the sensitivity and specificity of LAMP with PCR, normal PCR reactions were performed using the *bla*_OXA-51-like-F_ and *bla*_OXA-51-like-R_ primers (**Table 2**). The primers were synthesized commercially by Sangon Biotech Co., Ltd. (Beijing, China). PCR was performed as described previously (Kuo et al., 2012).

### PCR Detection of Carbapenemase Genes

The class D OXA carbapenemases of *Acinetobacter* sp. are represented by 4 main phylogenetic subgroups: OXA-23-like, OXA-24-like, OXA-51-like, and OXA-58. The primers used for detecting these genes by PCR are listed in **Table 2**. PCR was performed in a 25-μl reaction mixtures containing 12.5 μl PCR Master Mix Reagent (Tiangen Biotech Co., Ltd., Beijing, China); 1 μl of forward primer (10 pmol), 1 μl of reverse primer (10 pmol), and 1 μl of DNA template. Reaction mixtures were initially heated to 94°C for 5 min, followed by 30 cycles at 94°C for 30 s, 55°C for 30 s, and 72°C for 90 s. The final extension step was performed at 72°C for 7 min. The PCR-amplified products were analyzed by 1% agarose gel electrophoresis and stained with ethidium bromide. Images were acquired using a Bio-Rad Gel Doc EQ imaging system.

## Results

### Optimization of the LAMP Assay Targeting *bla*_OXA-51-like_

**FIGURE 1:**
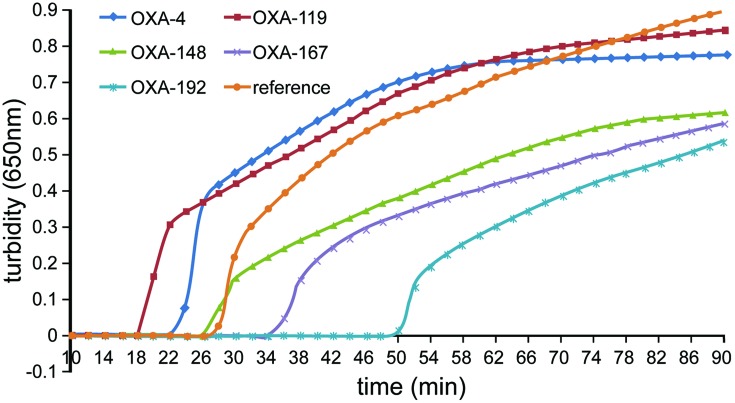
**Five sets of primers were used to amplify the indicated target genes of *Acinetobacter baumannii* under the same conditions**. Turbidity was monitored using a Loopamp real-time turbidimeter by measuring the absorbance at 650 nm every 6 s. The OXA-4 primer set was chosen as the most appropriate primers for the rapid detection of *A. baumannii.*

Five sets of different primers were initially tested for detection of the *bla*_OXA-51-like_ gene, and the primers for *pga*D gene of *A. baumannii* were added as a control (Wang et al., 2013). Four of the five primer sets enabled successful amplification (**Figure 1**). The OXA-4 primer set amplified the target sequence within the shortest time and was, therefore, chosen as the optimal primer set (**Table 2**).

**FIGURE 2:**
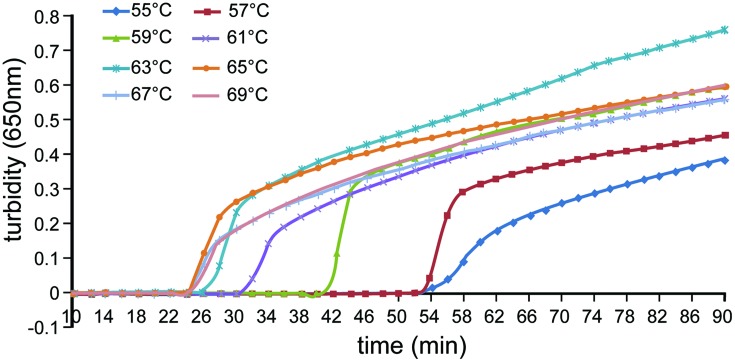
**Effect of differing temperatures on the efficiency of detection of *A. baumannii* by loop-mediated isothermal amplification (LAMP)**. Turbidity was monitored using a Loopamp real-time turbidimeter by measuring the absorbance at 650 nm every 6 s.

Reaction temperatures ranging from 55 to 69°C at a 2°C intervals were compared to determine optimal amplification conditions. The amplification efficiency was highest at 65°C (**Figure 2**) and was, therefore, used in subsequent experiments.

### Specificity and Sensitivity of the LAMP Assay

**FIGURE 3:**
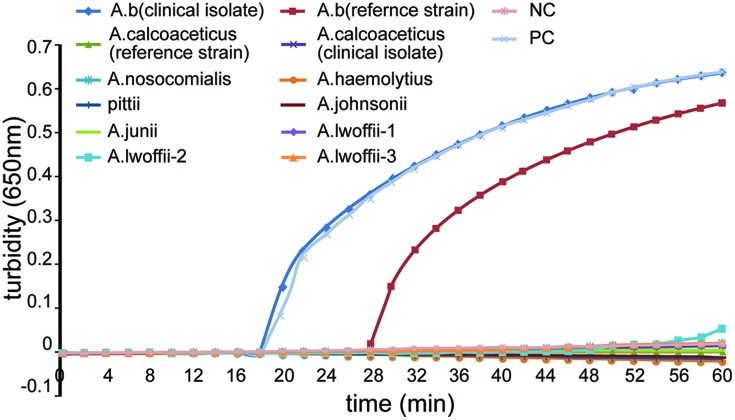
**Specificity of the LAMP reactions in detecting the *bla*_OXA-51-like_ gene**. Turbidity was monitored using a Loopamp real-time turbidimeter by measuring the absorbance at 650 nm every 6 s. Amplification was performed at 65°C for 60 min.

To evaluate the specificity of LAMP detection for *A. baumannii*, genomic DNA was extracted from 2 *A. baumannii* strains, as well as other 7 *Acinetobacter* species (10 strains) and 22 non-*A. baumannii* reference strains, and tested using real-time turbidity or visual detection of color changes as readouts. Genomic *A. baumannii* DNA (ATCC 22933) and distilled water were used as positive and negative controls, respectively. As shown in **Figure 3**, both methods of analysis positively identified the *A. baumannii* isolates. All other strains (as well as the blank control) tested negatively, indicating that the LAMP assay was specific for *A. baumannii*. Interestingly, the LAMP assay could differentiate *A. baumannii* from other *Acinetobacter* species.

**FIGURE 4:**
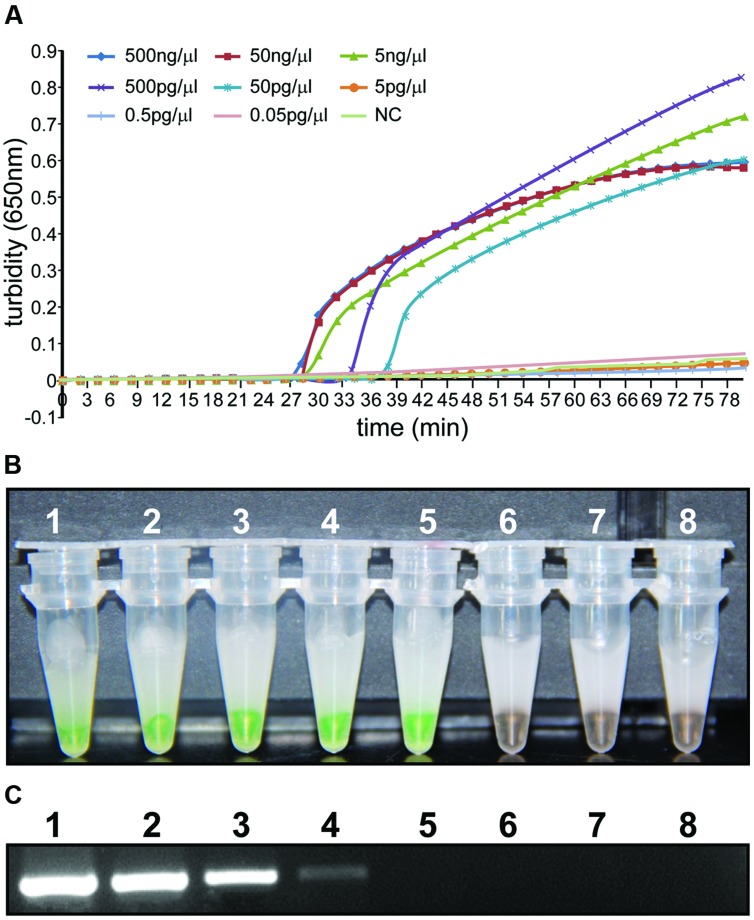
**Sensitivity of the LAMP reaction and PCR for detection of the *bla*_OXA-51-like_ gene**. Genomic DNA was diluted in a set of serial 10-fold dilutions. Both LAMP reactions **(A,B)** and PCR **(C)** were performed in duplicate for each dilution. Tubes and lanes: 1: 500 ng/μl, 2: 50 ng/μl, 3: 5 ng/μl, 4: 500 pg/μl, 5: 50 pg/μl, 6: 5 pg/μl, 7: 0.5 pg/μl, 8: 0.05 pg/μl. **(A)** Turbidity was monitored using a Loopamp real-time turbidimeter by measuring the absorbance at 650 nm every 6 s. **(B)** The direct visual method for the detection of LAMP. One microliter of fluorescent detection reagent was added to 25 μl of LAMP reaction mixture before the LAMP reactions were initiated. **(C)** PCR products were separated by 1% agarose gel electrophoresis and stained with ethidium bromide.

To compare the detection limit of traditional PCR with that of LAMP using either real-time turbidity or color-change measurements, 10-fold serial dilutions (50 ng/μl–5 pg/μl) were tested using genomic DNA extracted from *A. baumannii* ATCC 22933. As shown in **Figure 4**, the detection limits of real-time turbidity and visual detection were both 50 pg/μl, which was 10-fold more sensitive than traditional PCR assay.

### Detection of *A. baumannii* in Clinical Samples

A total of 355 clinical sputum samples and nasopharyngeal swabs were collected for LAMP-based surveillance of *bla*_OXA-51-like_ from ICU patients suspected of having multidrug-resistant infections in two hospitals in Beijing, China. Ten pairs of sputum samples and nasopharyngeal swabs from healthy people were collected as controls. All clinical samples were simultaneously analyzed by LAMP and PCR. Of the 355 clinical samples, the LAMP assay detected 228 positive samples and 127 negative samples, while the PCR assay detected 221 positive samples, and 134 negative samples. Then, *A. baumannii* was successfully cultured from all 228 samples that were positively identified by LAMP. Samples from the healthy control subjects all tested negatively by LAMP and PCR. The LAMP assay showed 100% specificity compared to 91.89% by PCR assay. Thus, the results showed the LAMP assays were more sensitive and specific than PCR for the diagnosis of *A. baumannii* in clinical samples.

### Antibiotic Susceptibility and Oxacillinase Distribution

A total of 228 clinical *A. baumannii* isolates were characterized by antibiotic susceptibility testing using both the VITEK^®^2 system and disk diffusion. Resistance rates were very high, with nearly 90% isolates being resistant to ampicillin, 68.2% being MDR-AB, 66.5% being resistant to IPM, and 63.6% being resistant to MPM.

Among all 145 isolates resistant to both IPM and MPM, 100% harbored the *bla*_OXA-51-like_ gene, 92.3% (134 strains) co-occurred with the *bla*_OXA-23_ gene, 1 isolate carrying both the *bla*_OXA-51-like_ gene and *bla*_OXA-58_ gene, and no isolate carried the *bla*_OXA-24_ gene. Ten isolates were negative for the tested plasmid-mediated OXA-carbapenemase genes.

We also investigated the presence of the *bla*_OXA-51-like_ gene in *A. baumannii* isolates susceptible to carbapenem treatment. All of these isolates harbored the naturally occurring *bla*_OXA-51-like_ gene, making it an excellent candidate for the identification of *Acinetobacter* species.

### Molecular Epidemiology of Carbapenem-Resistant *A. baumannii*

Forty-one out of 145 carbapenem-resistant *A. baumannii* isolates were analyzed by PFGE. Genomic DNA digested with *Apa*I showing similar patterns were designated as the same type, and those with 1–3 different bands compared with this type were designated as subtypes. Digested DNA with >3 different bands compared with one type were designated as other types. Bionumerisc software V6.01 was used to perform cluster analysis of these data.

**FIGURE 5:**
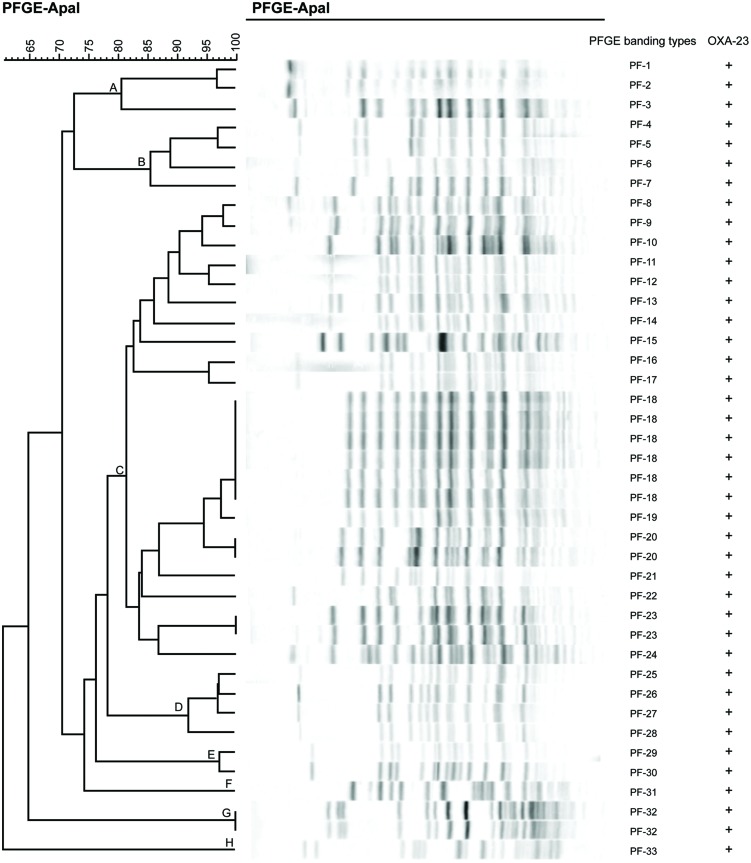
**Pulsed-field gel electrophoresis (PFGE) of *Apa* I-digested DNA from 41 *A. baumannii* isolates**. The dendrogram shown was generated via the unweighted pair-group method using arithmetic averages approach, based on Dice similarity coefficients that were determined using BioNumerics software, V6.01.

A total of 33 PFGE-banding types were identified, suggesting a diverse population of *A. baumannii* rather than the spread of a specific clone. According to the dice similarity index (80%), these types could be clustered into eight distinct PFGE patterns (clones A–H), and clone C was the dominant clone with 24 isolates (**Figure 5**). Clinical isolates with PFGE-banding type 18 (PF-18) represented the dominant epidemic strain in the ICU during the testing period.

## Discussion

Carbapenems including IPM and MPM are used as a last resort for treating MDR-AB. The incidence of carbapenem resistance in *A. baumannii* has increased steadily over the past decade and now represents a global problem. The Meropenem Yearly Susceptibility Test Information Collection program revealed a considerable worldwide increase in IPM and MPM resistance rates, which increased from 10 and 35% in 1999 to 47.9 and 57.4% in 2008, respectively (Rhomberg and Jones, 2009). Similarly, the SENTRY program documented an overall increase in IPM resistance from 34.5% in 2006 to 59.8% in 2009 (Gales et al., 2011). In the USA and Europe, carbapenem resistance accounted for 65% of *A. baumannii*-related pneumonia in 2012 (Farrell et al., 2014). In Asia, more than 60% of *A. baumannii* isolates causing hospital-acquired pneumonia were pan-resistant bacteria and resistant to carbapenem (Zarrilli et al., 2013; Tiwari and Moganty, 2014).

In this study, we observed that 66.5% (152 strains) or 63.6% (145 strains) of test isolates were resistant to IPM or MPM, respectively. The 228 clinical *A. baumannii* isolates were mainly collected from the ICUs of two hospitals; thus, the incidence of carbapenem resistance in *A. baumannii* was relatively high. Prolonged hospitalization, invasive medical procedures, and the prior use of broad-spectrum antibiotics (third-generation cephalosporins and fluoroquinolones) are risk factors for the acquisition of MDR-AB (Karageorgopoulos and Falagas, 2008). These findings indicated that treatment with carbapenem may potentially induce carbapenem resistance.

During PFGE analysis, 33 PFGE-banding patterns were identified and classified into 8 distinct clones, based on Tenover’s criteria (Tenover et al., 1995). Clone C was the dominant clone, making it possibly responsible for the *A. baumannii* epidemic in the ICUs of the two comprehensive hospitals from which we obtained the isolates. Clinical isolates of subtype PF-18 represented the main epidemic strain during the testing period. Previous results have demonstrated that carbapenem-resistant *A. baumannii* isolates showing the same PFGE patterns possess the same carbapenemase-associated genes (Yan et al., 2010; Park et al., 2012); however, our results were not consistent with these observations. Three isolates carrying only the *bla*_OXA-51-like_ gene, but not the *bla*_OXA-23_ gene, were grouped into different clones. However, isolates carrying both the *bla*_OXA-51-like_ gene and the *bla*_OXA-23_ gene were clustered into distinct clones, either. This may be due to the complicated resistance mechanism that underlies carbapenem resistance in *A. baumannii*.

Carbapenem susceptibility in *A. baumannii* is compromised by a variety of mechanisms. By far the most common mechanism are carbapenemases (Pogue et al., 2013). Carbapenem-hydrolyzing class D β-lactamases (CHDLs, Ambler class D), also known as oxacillinases, are the most common mediator of carbapenem resistance in *A. baumannii* (Poirel and Nordmann, 2006), although they are relatively inefficient compared with other types of carbapenemases, such as class B metallo-β-lactamases (MBLs; Ambler class B) represented by the VIM, IMP, SIM families, and the recently discovered New Delhi metallo-β-lactamase (NDM).

Among CHDLs in *A. baumannii*, there are four major distinct groups, including OXA-23, OXA-24, OXA-51-like, and OXA-58, which are encoded by the *bla*_OXA-23_, *bla*_OXA-24_, *bla*_OXA-51-like_, and *bla*_OXA-58_ genes, respectively (Poirel and Nordmann, 2006; Zavascki et al., 2010). The *bla*_OXA-23_ gene has been reported in *A. baumannii* isolates worldwide and is mediated both by chromosomal integration and plasmids. The *bla*_OXA-24_ gene can also be localized to chromosomes or plasmids, appears to be less widespread than the *bla*_OXA-23_ gene, and is relatively rare in China (Poirel et al., 2010). The chromosomally located *bla*_OXA-51-like_ gene is unique and naturally occurring in *A. baumannii*; thus, this gene is becoming an important genetic marker for the identification of organisms at the species level (Turton et al., 2006,). The plasmid-mediated *bla*_OXA-58_ gene has been reported in *Acinetobacter* sp. from many countries around the world, but is rare in China.

In this research, we found that in all the 145 CRAB isolates, the co-occurrence of *bla*_OXA-51-like_ gene and *bla*_OXA-23_ gene could be detected in 134 isolates (92.3%). This result indicated that the plasmid-mediated transfer of *bla*_OXA-23_ gene might be the most common mechanism by which *A. baumannii* becomes carbapenem resistance in these two comprehensive military hospitals in Beijing. Although the *bla*_OXA-58_ gene and *bla*_OXA-24_ gene have been reported in many countries, but the prevalence of these two genes is relatively rare in China. That could be explained that only one isolate carried the *bla*_OXA-58_ gene and no *bla*_OXA-24_ gene was detected in the CRAB isolates. However, 10 isolates could detect none of the plasmid-mediated OXA-carbapenemase genes including *bla*_OXA-23,_ *bla*_OXA-24_ and *bla*_OXA-58,_ which indicated that these isolates might harbored other resistant genes such as MBLs against carbapenem. These data are consistent with other reports from China and other countries (Turton et al., 2006; Woodford et al., 2006; Zavascki et al., 2010; Tiwari et al., 2012a).

In the conventional PCR assay, the *bla*_OXA-51-like_ gene could not be amplified from seven clinical isolates. We thought that the low bacterial contents and incorrect pathogen identification may account for the failure to amplify these samples. Thus, the seven strains were analyzed for the presence of the *bla*_OXA-51-like_ gene by the LAMP method described here, yielding positive results in each case, which was confirmed using the Vitek 2 system. Our results showed that these clinical isolates were actually *A. baumannii*. These results suggest that occasionally the sensitivity of conventional PCR is not satisfactory and may introduce errors into diagnostic test results.

Compared with traditional methods for pathogen diagnosis, LAMP assays can generate results more easily and rapidly, with high sensitivity and specificity (Notomi et al., 2000). The LAMP reaction does not require temperature cycling, with just a temperature-controlled water bath or a constant-temperature environment being sufficient. LAMP primers are designed to match four or six of the six or eight independent target-sequence regions, so the specificity and sensitivity are enhanced greatly. Under optimum conditions, the LAMP reaction can be completed in 1 h. In this study, we described a LAMP assay based on the *bla*_OXA-51-like_ gene for detecting *A. baumannii*. This LAMP method could detect the target DNA within 60 min at an isothermal temperature of 65°C. The detection limit of the LAMP assay was 50 pg/μl, which was about 10-fold greater than that of PCR. Furthermore, the LAMP method described here could distinguish *A. baumannii* from *A. nosocomialis* (genospecies 13TU), *A. pittii* (genospecies 3), and the *A. Baumannii* complex (*A. baumannii*, *A. nosocomialis*, *A. pittii*, and *A. calcoaceticus*). Previously, there were two reports on the use of an *A. baumannii* LAMP assay, based on targeting the 16S–23S rRNA intergenic spacer sequence (Soo et al., 2013) and the conserved regions of the *pga*D gene of *A. baumannii* (Wang et al., 2013). However, the former could not effectively distinguish *A. baumannii* from *A. nosocomialis*, and *A. pittii*, and the specificity of *A. baumannii* LAMP assay of the latter was only 75%.

## Conclusion

A rapid, sensitive, specific, and effective LAMP assay was established for the detection of *A. baumannii*. The LAMP assay will be very useful for the rapid detection of pathogens in clinical samples. Meanwhile, this report provides some therapeutic recommendations for the treatment of *A. baumannii* infection and a warning that the growing emergence of carbapenem-resistant *A. baumannii* has led to an inadequacy of therapeutic choices in treating MDR-AB infections among patients in China. Our findings further emphasize that when investigating outbreaks caused by carbapenem-resistant *A. baumannii*, both the detection of carbapenemase-associated genes and PFGE are needed.

## Author Contributions

Puyuan Li and Wenkai Niu wrote the main manuscript text. Puyuan Li, Huan Li, and Wei Liu prepared **Figures 1–4**. Leijing Guo prepared **Figure 5**. Puyuan Li, Wenkai Niu, Hong Lei, Xiangna Zhao, Dayang Zou, Xin Yuan, and Huiying Liu executed the experiments. Changqing Bai and Jing Yuan helped conceive the project and designed the experiments. All authors reviewed the manuscript.

## Conflict of Interest Statement

The authors declare that the research was conducted in the absence of any commercial or financial relationships that could be construed as a potential conflict of interest.

## References

[B1] Álvarez-Buylla A. Culebras E. Picazo J. J. (2012). Identification of *Acinetobacter* species: is Bruker biotyper MALDI-TOF mass spectrometry a good alternative to molecular techniques? *Infect. Genet. Evol.* 12 345–349. 10.1016/j.meegid.2012.01.002 22266021

[B2] Antunes L. Visca P. Towner K. J. (2014). *Acinetobacter baumannii*: evolution of a global pathogen. *Pathog. Dis.* 71 292–301. 10.1111/2049-632X.12125 24376225

[B3] Chiang M.-C. Kuo S.-C. Chen S.-J. Yang S.-P. Lee Y.-T. Chen T.-L. (2012). Clinical characteristics and outcomes of bacteremia due to different genomic species of *Acinetobacter baumannii* complex in patients with solid tumors. *Infection* 40 19–26. 10.1007/s15010-011-0187-4 21887526

[B4] Dijkshoorn L. Nemec A. Seifert H. (2007). An increasing threat in hospitals: multidrug-resistant *Acinetobacter baumannii*. *Nat. Rev. Microbiol.* 5 939–951. 10.1038/nrmicro1789 18007677

[B5] Dinh D. T. Le M. T. Q. Vuong C. D. Hasebe F. Morita K. (2011). An updated loop-mediated isothermal amplification method for rapid diagnosis of H5N1 Avian Influenza Viruses. *Trop. Med. Health* 39 3–7. 10.2149/tmh.2010-21 22028606 PMC3153145

[B6] Farrell D. J. Sader H. S. Flamm R. K. Jones R. N. (2014). Ceftolozane/tazobactam activity tested against Gram-negative bacterial isolates from hospitalised patients with pneumonia in US and European medical centres (2012). *Int. J. Antimicrob. Agents* 43 533–539. 10.1016/j.ijantimicag.2014.01.032 24856078

[B7] Gales A. C. Jones R. N. Sader H. S. (2011). Contemporary activity of colistin and polymyxin B against a worldwide collection of Gram-negative pathogens: results from the SENTRY Antimicrobial Surveillance Program (2006–09). *J. Antimicrob. Chemother.* 66 2070–2074. 10.1093/jac/dkr239 21715434

[B8] Golanbar G. D. Lam C. K. Chu Y.-M. Cueva C. Tan S. W. Silva I. (2011). Phenotypic and molecular characterization of *Acinetobacter* clinical isolates obtained from inmates of California correctional facilities. *J. Clin. Microbiol.* 49 2121–2131. 10.1093/jac/dkr239 21450955 PMC3122719

[B9] Gotoh K. Nishimura N. Takeuchi S. Hattori F. Horiba K. Isaji M. (2013). Assessment of the loop-mediated isothermal amplification assay for rapid diagnosis of *Mycoplasma pneumoniae* in pediatric community-acquired pneumonia. *Jpn. J. Infect. Dis.* 66 539–542. 10.1007/s10156-012-0388-5 24270147

[B10] Janda J. M. Abbott S. L. (2007). 16S rRNA gene sequencing for bacterial identification in the diagnostic laboratory: pluses, perils, and pitfalls. *J. Clin. Microbiol.* 45 2761–2764. 10.1128/jcm.01228-07 17626177 PMC2045242

[B11] Karageorgopoulos D. E. Falagas M. E. (2008). Current control and treatment of multidrug-resistant *Acinetobacter baumannii* infections. *Lancet Infect. Dis.* 8 751–762. 10.1016/S1473-3099(08)70279-2 19022191

[B12] Karah N. Haldorsen B. Hegstad K. Simonsen G. S. Sundsfjord A. Samuelsen Ø (2011). Species identification and molecular characterization of *Acinetobacter* spp. blood culture isolates from Norway. *J. Antimicrob. Chemother.* 66 738–744. 10.1093/jac/dkq521 21393175

[B13] Kempf M. Rolain J.-M. (2012). Emergence of resistance to carbapenems in *Acinetobacter baumannii* in Europe: clinical impact and therapeutic options. *Int. J. Antimicrob. Agents* 39 105–114. 10.1016/j.ijantimicag.2011.10.004 22113193

[B14] Kumar P. Pandya D. Singh N. Behera D. Aggarwal P. Singh S. (2014). Loop-mediated isothermal amplification assay for rapid and sensitive diagnosis of tuberculosis. *J. Infect.* 69 607–615. 10.1016/j.jinf.2014.08.017 25218428

[B15] Kuo H. Y. Chang K. C. Kuo J. W. Yueh H. W. Liou M. L. (2012). Imipenem: a potent inducer of multidrug resistance in *Acinetobacter baumannii*. *Int. J. Antimicrob. Agents* 39 33–38. 10.1016/j.ijantimicag.2011.08.016 21996406

[B16] Lemos E. de la Hoz F. Einarson T. McGhan W. Quevedo E. Castañeda C. (2014). Carbapenem resistance and mortality in patients with *Acinetobacter baumannii* infection: systematic review and meta-analysis. *Clin. Microbiol. Infect.* 20 416–423. 10.1111/1469-0691.12363 24131374

[B17] Nakauchi M. Takayama I. Takahashi H. Tashiro M. Kageyama T. (2014). Development of a reverse transcription loop-mediated isothermal amplification assay for the rapid diagnosis of avian influenza A (H7N9) virus infection. *J. Virol. Methods* 204 101–104. 10.1016/j.jviromet.2014.03.028 24747008

[B18] Nakauchi M. Yoshikawa T. Nakai H. Sugata K. Yoshikawa A. Asano Y. (2011). Evaluation of reverse transcription loop-mediated isothermal amplification assays for rapid diagnosis of pandemic influenza A/H1N1 2009 virus. *J. Med. Virol.* 83 10–15. 10.1002/jmv.21934 21108334

[B19] Notomi T. Okayama H. Masubuchi H. Yonekawa T. Watanabe K. Amino N. (2000). Loop-mediated isothermal amplification of DNA. *Nucleic Acids Res.* 28 E63 10.1093/nar/28.12.e63 PMC102748 10871386

[B20] Olive D. M. Bean P. (1999). Principles and applications of methods for DNA-based typing of microbial organisms. *J. Clin. Microbiol.* 37 1661–1669. 10325304 10.1128/jcm.37.6.1661-1669.1999 PMC84917

[B21] Park Y. K. Jung S.-I. Park K.-H. Kim S. H. Ko K. S. (2012). Characteristics of carbapenem-resistant *Acinetobacter* spp. other than *Acinetobacter baumannii* in South Korea. *Int. J. Antimicrob. Agents* 39 81–85. 10.1016/j.ijantimicag.2011.08.006 21996405

[B22] Peleg A. Y. Seifert H. Paterson D. L. (2008). *Acinetobacter baumannii*: emergence of a successful pathogen. *Clin. Microbiol. Rev.* 21 538–582. 10.1128/CMR.00058-07 18625687 PMC2493088

[B23] Perez F. Hujer A. M. Hujer K. M. Decker B. K. Rather P. N. Bonomo R. A. (2007). Global challenge of multidrug-resistant *Acinetobacter baumannii*. *Antimicrob. Agents Chemother.* 51 3471–3484. 10.1128/aac.01464-06 17646423 PMC2043292

[B24] Pogue J. M. Mann T. Barber K. E. Kaye K. S. (2013). Carbapenem-resistant *Acinetobacter baumannii*: epidemiology, surveillance and management. *Expert Rev. Anti Infect. Ther.* 11 383–393. 10.1586/eri.13.14 23566148

[B25] Poirel L. Naas T. Nordmann P. (2010). Diversity, epidemiology, and genetics of class D beta-lactamases. *Antimicrob. Agents Chemother.* 54 24–38. 10.1128/AAC.01512-08 19721065 PMC2798486

[B26] Poirel L. Nordmann P. (2006). Carbapenem resistance in *Acinetobacter baumannii*: mechanisms and epidemiology. *Clin. Microbiol. Infect.* 12 826–836. 10.1111/j.1469-0691.2006.01456.x 16882287

[B27] Poon L. L. Leung C. S. Tashiro M. Chan K. H. Wong B. W. Yuen K. Y. (2004). Rapid detection of the severe acute respiratory syndrome (SARS) coronavirus by a loop-mediated isothermal amplification assay. *Clin. Chem.* 50 1050–1052. 10.1373/clinchem.2004.032011 15054079 PMC7108160

[B28] Rhomberg P. R. Jones R. N. (2009). Summary trends for the meropenem yearly susceptibility test information collection program: a 10-year experience in the United States (1999–2008). *Diagn. Microbiol. Infect. Dis.* 65 414–426. 10.1016/j.diagmicrobio.2009.08.020 19833471

[B29] Soo P.-C. Tseng C.-C. Ling S.-R. Liou M.-L. Liu C.-C. Chao H.-J. (2013). Rapid and sensitive detection of *Acinetobacter baumannii* using loop-mediated isothermal amplification. *J. Microbiol. Methods* 92 197–200. 10.1016/j.mimet.2012.11.020 23220188

[B30] Tenover F. C. Arbeit R. D. Goering R. V. Mickelsen P. A. Murray B. E. Persing D. H. (1995). Interpreting chromosomal DNA restriction patterns produced by pulsed-field gel electrophoresis: criteria for bacterial strain typing. *J. Clin. Microbiol.* 33 2233–2239. 7494007 10.1128/jcm.33.9.2233-2239.1995 PMC228385

[B31] Tiwari V. Kapil A. Moganty R. R. (2012a). Carbapenem-hydrolyzing oxacillinase in high resistant strains of *Acinetobacter baumannii* isolated from India. *Microb. Pathog.* 53 81–86. 10.1016/j.micpath.2012.05.004 22610043

[B32] Tiwari V. Vashistt J. Kapil A. Moganty R. R. (2012b). Comparative proteomics of inner membrane fraction from carbapenem-resistant *Acinetobacter baumannii* with a reference strain. *PLoS ONE* 7:e39451 10.1371/journal.pone.0039451 PMC3383706 22761799

[B33] Tiwari V. Moganty R. R. (2014). Conformational stability of OXA-51 β-lactamase explains its role in carbapenem resistance of *Acinetobacter baumannii*. *J. Biomol. Struct. Dyn.* 32 1406–1420. 10.1080/07391102.2013.819789 23879430

[B34] Turton J. F. Woodford N. Glover J. Yarde S. Kaufmann M. E. Pitt T. L. (2006). Identification of *Acinetobacter baumannii* by detection of the blaOXA-51-like carbapenemase gene intrinsic to this species. *J. Clin. Microbiol.* 44 2974–2976. 10.1128/jcm.01021-06 16891520 PMC1594603

[B35] Wang Q. Zhou Y. Li S. Zhuo C. Xu S. Huang L. (2013). Real-time fluorescence loop mediated isothermal amplification for the detection of *Acinetobacter baumannii*. *PLoS ONE* 8:e66406 10.1371/journal.pone.0066406 PMC3699609 23843955

[B36] Woodford N. Ellington M. J. Coelho J. M. Turton J. F. Ward M. E. Brown S. (2006). Multiplex PCR for genes encoding prevalent OXA carbapenemases in *Acinetobacter* spp. *Int. J. Antimicrob. Agents* 27 351–353. 10.1016/j.ijantimicag.2006.01.004 16564159

[B37] Xi H. Xu Y. Zhu D. Wang F. Ni Y. Sun J. (2012). CHINET 2010 surveillance of antibiotic resistance in *Acinetobacter baumannii* in China. *Chin. J. Infect. Chemother.* 2 006.

[B38] Yan Z.-Q. Shen D.-X. Cao J.-R. Chen R. Wei X. Liu L.-P. (2010). Susceptibility patterns and molecular epidemiology of multidrug-resistant *Acinetobacter baumannii* strains from three military hospitals in China. I. *J. Antimicrob. Agents* 35 269–273. 10.1016/j.ijantimicag.2009.10.016 20036519

[B39] Zarrilli R. Pournaras S. Giannouli M. Tsakris A. (2013). Global evolution of multidrug-resistant *Acinetobacter baumannii* clonal lineages. *Int. J. Antimicrob. Agents* 41 11–19. 10.1016/j.ijantimicag.2012.09.008 23127486

[B40] Zavascki A. P. Carvalhaes C. G. Picão R. C. Gales A. C. (2010). Multidrug-resistant *Pseudomonas aeruginosa* and *Acinetobacter baumannii*: resistance mechanisms and implications for therapy. *Expert Rev. Anti Infect. Ther.* 8 71–93. 10.1586/eri.09.108 20014903

[B41] Zeng Y. Zhang X. Nie K. Ding X. Ring B. Z. Xu L. (2014). Rapid quantitative detection of Human immunodeficiency virus type 1 by a reverse transcription-loop-mediated isothermal amplification assay. *Gene* 541 123–128. 10.1016/j.gene.2014.03.015 24630968

